# Targeting the Ischemic Core: A Therapeutic Microdialytic Approach to Prevent Neuronal Death and Restore Functional Behaviors

**DOI:** 10.3390/ijms26083821

**Published:** 2025-04-17

**Authors:** May-Jywan Tsai, Dann-Ying Liou, Li-Yu Fay, Shih-Ling Huang, Wen-Cheng Huang, Chang-Ming Chern, Shen-Kou Tsai, Henrich Cheng, Shiang-Suo Huang

**Affiliations:** 1 Department of Neurosurgery, Neurological Institute, Taipei Veterans General Hospital, Taipei 11217, Taiwan; 2 Division of Neural Regeneration and Repair, Neurological Institute, Taipei Veterans General Hospital, Taipei 11217, Taiwan; 3 Department of Medicine, National Yang Ming Chiao Tung University, Taipei 11230, Taiwan; 4 Department of Neurology, Neurological Institute, Taipei Veterans General Hospital, Taipei 11217, Taiwan; 5 Department of Neurology, En Chu Kong Hospital, New Taipei City 23702, Taiwan; 6 Department of Anesthesiology, Cheng Hsin General Hospital, Taipei 11283, Taiwan; 7 Department and Institute of Pharmacology, National Yang Ming Chiao Tung University, Taipei 11230, Taiwan; 8 Department of Pharmacology and Institute of Medicine, Chung Shan Medical University, Taichung 40201, Taiwan; 9 Department of Pharmacy, Chung Shan Medical University Hospital, Taichung 40201, Taiwan

**Keywords:** microdialysis, neuroprotection, serum albumin, cerebral ischemia

## Abstract

Ischemic stroke leads to cerebral ionic imbalance, increases acidosis, oxidative stress and release of glutamate and inflammatory mediators. Removing solute or stimulants from the ischemic core may block cell-damaging events and confer neuroprotection. In this study, we developed a minimally invasive therapeutic microdialysis (tMD) method, choosing to include serum albumin in the buffer because it is a multifunctional protein with osmotic properties. Aiming at the ischemic core, continuous perfusion of buffer supplemented with osmotic agents removes mediators of inflammation/cell damage/death from the lesion. This tMD treatment significantly removed the glutamate and zinc ions from the core, thereby reducing infarct volumes and affording high-grade neurobehavioral protection against ischemic stroke. The tMD treatment effectively protected neurons and reduced microglial activation. Furthermore, this tMD approach extended the therapeutic window to protect beyond 6 h after stroke onset. These findings support the potential clinical feasibility of applying tMD to patients with ischemic stroke, potentially without adverse effects.

## 1. Introduction

Stroke is caused by a focal or global insufficiency of blood flow to the brain [1] and has been the second leading cause of death worldwide for an extended period [2,3]. More than 80% of stroke patients experience cerebral ischemia [4]. After ischemic stroke, two major regions of damage in the brain can be defined according to the remaining blood supply. The core of the insult is complete obstruction of blood supply and almost complete energetic failure resulting in necrosis. The penumbra is the area surrounding the ischemic core with mild to moderate reductions in cerebral blood flow during the occlusion period [4,5]. Clinicians intervene early in the penumbra as a target to rescue brain tissue and reduce post-stroke disability. Despite substantial research focusing on neuroprotective drug candidates, none have proven satisfactory for stroke treatment or have been successfully translated into clinical use in stroke patients [6]. Preclinical studies have identified several promising compounds, including resveratrol [7], melibiose [8], and melatonin [9], which exhibit neuroprotective, anti-inflammatory, and antioxidant properties. However, their efficacy in human stroke patients remains uncertain due to the lack of large-scale clinical trials. The current goal of treatment is to achieve reperfusion of the ischemic penumbra to protect the brain from further damage after stroke. This includes intra-arterial and intravenous thrombolysis, recombinant tissue plasminogen activator (rt-PA) [10], or mechanical thrombectomy [11]. Nowadays, the best effective treatment for acute ischemic stroke according to stroke guidelines is intravenous rt-PA within 4.5 h or intra-arterial mechanical thrombectomy within 24 h after stroke onset [12,13]. However, a substantial proportion of ischemic stroke patients face challenges in achieving timely or successful removal of the thrombus through intravascular thrombolysis, resulting in permanent central infarction. Even among patients who successfully undergo thrombolytic therapy, ischemia-reperfusion injury may still occur [14,15]. Thus, it is imperative to develop alternative restorative therapies that can be applied to the majority of stroke patients.

Ischemic stroke triggers multiple molecular events that lead to cerebral damage including excitotoxicity, reactive oxygen species production, inflammation, and cell death processes [16]. Removal of solute or stimulants from the ischemic core area may stop cell damaging events and confer neuroprotection. This study employs a breakthrough use of cerebral microdialysis (MD) as a therapeutic approach, aiming to intervene in the disastrous ischemic cascade. In this project, a minimally invasive MD probe was inserted into the brain parenchyma, targeting the ischemia core to remove mediators of inflammation/cell injury/death in the nidus. With supplementation of a beneficial agent in the perfusing buffer, the effect of therapeutic microdialysis (tMD) on cerebral ischemia was examined. Serum albumin was selected to be included in the buffer because it is a multifunctional protein with an oncotic property. The effects of human albumin have been demonstrated in models of acute cerebral ischemia [17,18]. Albumin therapy has been shown to offer neuroprotective effects in ischemic stroke by modulating immune and inflammatory responses. Studies have demonstrated that albumin administration can reduce Toll-like receptor 4 expression while increasing anti-inflammatory cytokines such as interleukin-10 and transforming growth factor beta1, leading to a reduction in immune inflammatory responses. Additionally, albumin increases the percentage of regulatory T cells (Tregs), further contributing to its protective effects [19]. Albumin therapy in acute ischemic stroke has shown potential benefits, but it is also associated with significant adverse effects, including pulmonary edema and increased mortality rates [20]. The ALIAS (Albumin in Acute Stroke) trials used 25% human serum albumin to investigate clinical outcomes in acute stroke patients when administered within 5 h of symptom onset. They found that albumin therapy did not significantly improve stroke outcomes and presented considerable risks, particularly pulmonary complications. In fact, pulmonary edema occurred in a notable percentage of patients, and higher mortality rates were observed in the albumin-treated groups [21]. These findings highlight that translating the benefits of albumin therapy into clinical practice remains challenging when administering intravenous albumin in stroke patients. Our tMD method, which retains serum albumin inside the microdialysis probe, is a novel and safe treatment modality and is not intended to provide supplemental albumin to the systemic circulation. Furthermore, we identified an unanticipated role for the albumin-supplemented MD buffer in the removal of glutamate and toxicants through the tMD method. Thus, our investigation introduces an unexplored approach to cerebral protection by reducing the post-stroke injury cascade and promoting functional recovery through direct removal of toxicants in the ischemic core.

## 2. Results

**Scheme 1:**
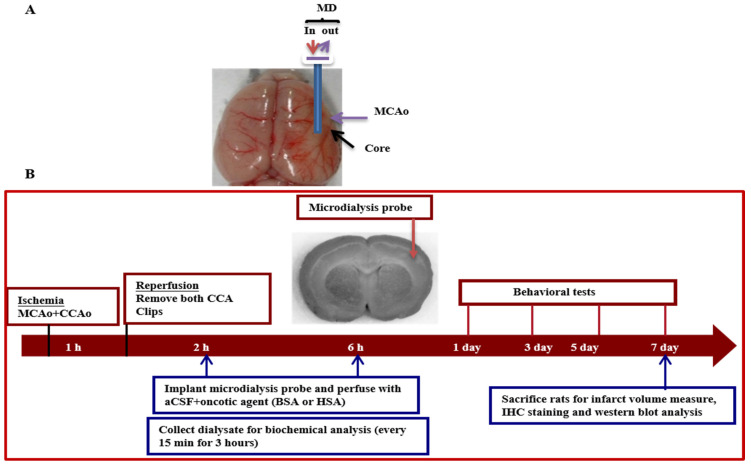
Protocol for application of therapeutic microdialysis (tMD) in ischemic rat brains after ischemia-reperfusion. (**A**) rat brain with a MD probe pre-equilibrated with buffer was inserted to the ischemic core. (**B**) A microdialysis probe pre-equilibrated with an aCSF-based dialytic buffer, was inserted into the ischemic core at 2 or 6 h after ischemic-reperfusion and microdialysis persisted for 3 h. Experimental rats were allowed to survive for one week. Rat brains were then removed for infarct volume analysis, morphology, and Western blot analysis.

Scheme 1 demonstrates the protocol for application of tMD in ischemic rat brains after ischemia-reperfusion. The scheme shows the three epochs (ischemia, reperfusion before treatment, and post-treatment) over which behavioral performance was scored. A microdialysis probe (with a 4 mm membrane window), pre-equilibrated with an aCSF-based dialytic buffer, was inserted into the ischemic core at 2 or 6 h after ischemic-reperfusion and persistent perfusion for 3 h. At one week post-injury and treatment, the experimental rat brains were collected for infarct volume analysis (using TTC staining) and for immunohistochemical or Western blot analysis.

### 2.1. Enhancement of Brain Damage and Motor Deficits by Stroke and Its Reduction by Microdialysis with Bovine Serum Albumin (BSA)

**Figure 1:**
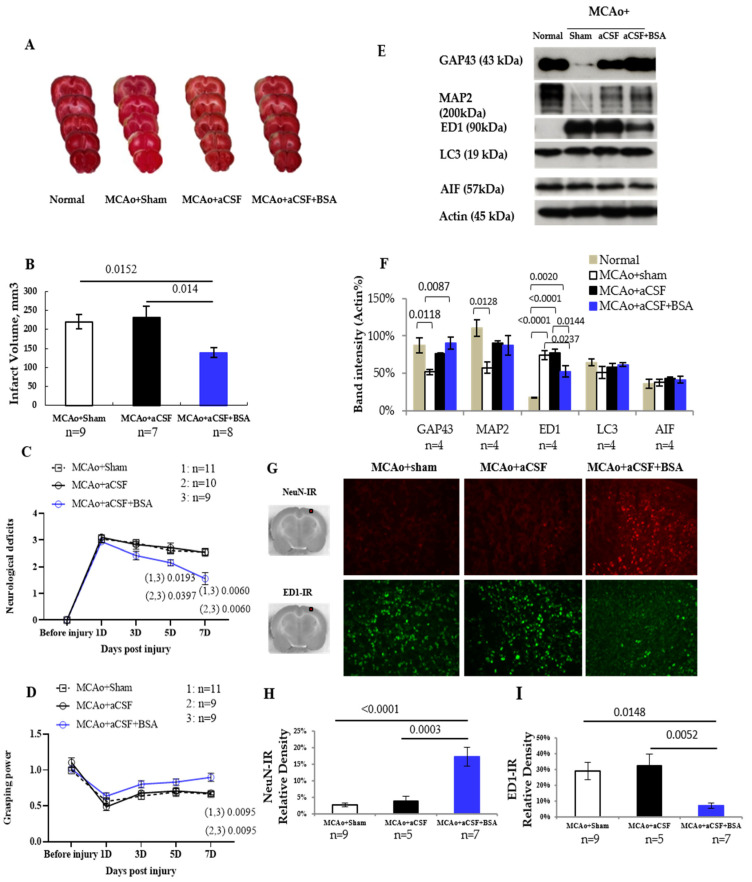
Effect of tMD at 2 h post-injury on brain infarction and behavioral performance in MCAo rats. (**A**) TTC staining of brain sections for measurement of infarct size after stroke and treatment. Negative TTC stained area denotes the infarct area in ischemic brains. (**B**) Brain infarcted volume (mm^3^) in ischemic brain tissues (n = 9, 7, 8 rats for sham, aCSF, aCSF+BSA, respectively); significance at *p* = 0.014, aCSF vs. aCSF+BSA; *p* = 0.0152, sham vs. aCSF+BSA. (**C**) Neurological deficit scores in rats surviving one week after cerebral ischemia (n = 11, 10, 9 rats for sham, aCSF, aCSF+BSA, respectively). Five days behavioral significance at *p* = 0.0193, sham vs. aCSF+BSA; *p* = 0.0397 aCSF vs. aCSF+BSA; 7 days behavioral significance at *p* = 0.006, sham vs. aCSF+BSA; *p* = 0.006, aCSF vs. aCSF+BSA; (**D**) grasping power, which was evaluated by grip test on right forelimbs (unaffected side) and left forelimbs (stroke-affected side) in MCAo rats (n = 11, 9, 9 rats for sham, aCSF, aCSF+BSA, respectively); 7 days behavioral significance at *p* = 0.0095, sham vs. aCSF+BSA; *p* = 0.0095, aCSF vs. aCSF+HSA. Behavioral data were analyzed by two-way ANOVA followed by Holm–Sidak’s test. (**E**) Western blot analysis of rat brain lysates at 1 week post-injury. (**F**) Quantification of blots, normalized to internal control (actin), for cell markers GAP43, MAP-2, and ED1; for autophagy-related processes LC3, and for apoptosis-inducing factor (AIF). (**G**) Representative micrographs of NeuN and ED1 immunoreactive (IR) cells for neurons and activated microglia, respectively, in mid-infarct cortex, indicated by a red square in the subfigure, at 1 week after MCAo. Scale bar: 100 μm. (**H**) Histogram for quantitation of NeuN-IR density of images in panel G from n = 9, 5, 7 rats for sham, aCSF, aCSF+BSA, respectively. Significance at *p* = 0.0003, aCSF vs. aCSF+BSA; *p* < 0.0001, sham vs. aCSF+BSA by one-way ANOVA followed by Holm–Sidak’s test. (**I**) Histogram for quantitation of ED1-IR density of images in panel G from n = 9, 5, 7 rats for sham, aCSF, aCSF + BSA, respectively. Significance at *p* = 0.0052, aCSF vs. aCSF+BSA; *p* < 0.0148, sham vs. aCSF+BSA by one-way ANOVA followed by Holm–Sidak’s test. A microdialysis probe was implanted in the ischemic nidus at 2 h post-injury, and microdialysis persisted for 3 h. Data are expressed as the mean ± SEM.

During the first few hours after stroke, molecular events in the penumbra mostly comprise the injury. Acute removal of solute or stimulants from the ischemic core area may stop cell damaging events and confer neuroprotection. To test this hypothesis, a microdialysis probe was implanted to target the ischemic core area at 2 h after focal ischemia/reperfusion in rats in an attempt to clean off the core. The effect of BSA supplemented in the microdialytic buffer on cerebral ischemia was examined. The detailed application of microdialytic treatment in MCAo rats is depicted in Scheme 1. The results show that rats subjected to microdialysis with supplementation of BSA (10%) in aCSF buffer exhibited a small infarct volume caused by a 60 min episode of MCAo based on TTC staining after focal ischemia/reperfusion in rats (*p* < 0.05; Figure 1A,B). Rats receiving microdialysis with aCSF buffer (MCAo+aCSF group) did not decrease infarct volumes, compared with sham animals (MCAo+sham group) at 7 days after inducing MCAo. Normal rats receiving sham surgery (normal+aCSF) did not exhibit brain infarction in the 7-day recovery period (Figure 1A). In addition to cortical infarction, functional behaviors were quantitatively tested in the experimental rats. Figure 1C,D show that MCAo induced behavioral impairment in rats as evidenced by increased neurological deficit scores (NDS) and decreased measures of grasping power. Neurological function was markedly impaired at 1 day after MCAo in untreated sham rats with a tendency to recover spontaneously thereafter. At one day after MCAo, there was no obvious difference in the NDS test among the three experimental groups. Then, neurological deficits in aCSF+BSA+microdialysis-treated rats were significantly ameliorated at 5 and 7 days after MCAo (*p* < 0.05 or 0.01, Figure 1C), compared to that of the MCAo+sham or MCAo+aCSF-treated rats. The results of the grasping power test, which measured the grip strength of contralateral (left) forelimbs, declined in all ischemic groups at one day after MCAo, compared with pre-MCAo operation (Figure 1D). The strength of grasping power in the left forelimb of the aCSF+BSA+microdialysis-treated rats was remarkably improved at 7 days after MCAo (*p* < 0.01), whereas this phenomenon did not occur in the sham- or aCSF-treated group. These data suggest that acute intervention with microdialysis supplementing BSA in aCSF buffer tMD could effectively reduce brain infarction and improve motor functional recovery in MCAo rats. The protein expression in the ipsilateral brain surrounding the ischemic core was analyzed in all rat groups at one week after stroke onset. Figure 1E,F show the gel and quantitative results of the Western blot analysis. The expression of GAP43, a “growth” or “plasticity” protein, was reduced by injury but was more preserved in aCSF+BSA-treated and aCSF-treated groups compared to the sham-treated group (*p* < 0.05). The expression level of MAP-2, a neuronal marker, was reduced by injury but was more preserved in the aCSF+BSA-treated and aCSF-treated groups compared to the sham-treated group (*p* < 0.05). In contrast, the expression level of ED1 (CD68), an activated microglia/macrophage marker, was significantly increased after cerebral ischemic injury (sham or aCSF-treated). Microdialysis with aCSF+BSA markedly reduced ED-1 protein levels (*p* < 0.05). No difference was observed in the expression levels of LC3 and AIF, which are markers for new axons, autophagy and apoptosis, respectively, among all treatment groups (Figure 1F). Histological assessment of cell survival and microglial activation/infiltration was further conducted in the ipsilateral ischemic rat brains. Following an ischemic stroke, decreased NeuN (+) neurons and increased ED1 (+) microglia/macrophages were evident within the ischemic peri-infarct/penumbral regions (Figure 1G). Only a few ED1-positive cells were observed in the ischemic cortex in the groups receiving aCSF+BSA treatment. NeuN-positive neurons were more preserved in the aCSF+BSA treatment groups compared to the sham- or aCSF-treated MCAo rat cortex. Quantitative assessment of IHC results demonstrated that MCAo animals treated with aCSF+BSA have significantly more preserved immunoreactivity (IR) for NeuN (Figure 1H) compared with that in sham or vehicle-treated animals (*p* < 0.01), indicating neuroprotective treatment. Furthermore, tMD treatment significantly reduced activation or infiltration of microglia/macrophage (Figure 1I) compared with sham or vehicle-treated animals, suggestive of a reduced inflammatory response.

### 2.2. HSA Is as Effective as BSA in Working as an Oncotic Agent for tMD in MCAo Rats

**Figure 2:**
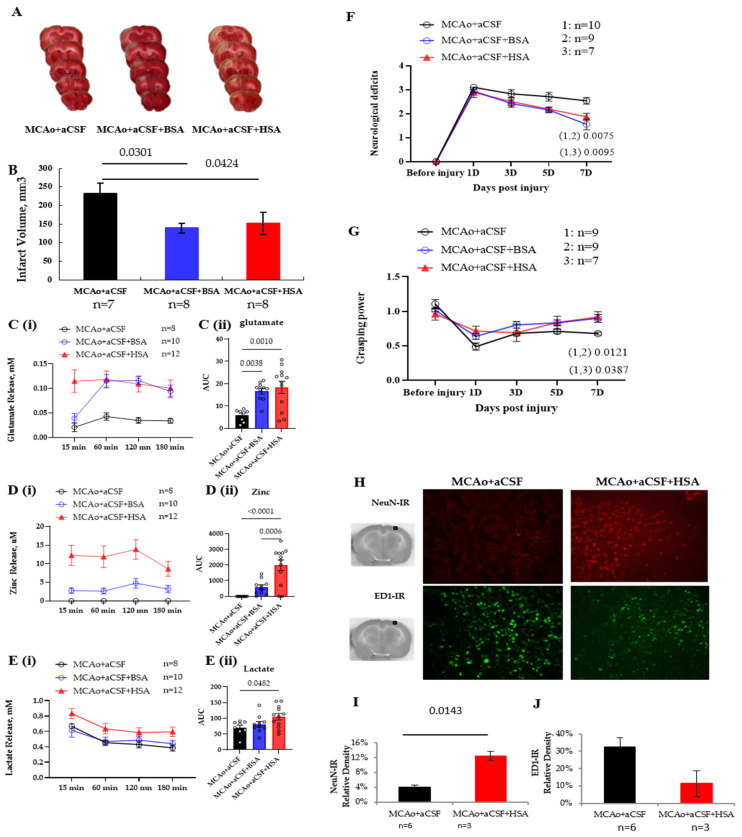
Human serum albumin (HSA) was as effective as BSA in working as an oncotic agent for tMD in MCAo rats. (**A**) Representative images of TTC staining of ischemic brain tissues after 1 week of reperfusion. (**B**) Infarcted volume (mm^3^) in ischemic brain tissues (n = 7, 8, 8 rats for aCSF, aCSF+BSA, aCSF+HSA, respectively); significance at *p* = 0.0301, aCSF vs. aCSF+BSA; *p* = 0.0424, aCSF vs. aCSF+HSA. (**C**) Glutamate levels in the microdialysate of MCAo rats between 2 and 5 h post-injury (n = 8, 10, 12 rats for aCSF, aCSF+BSA, aCSF+HSA, respectively). The change in glutamate levels in the microdialysate were plotted (line chart, left panel), and the area under the curve was analyzed (bar chart with symbol which shows individual data). Quantification of the area under curve (AUC) showing significance at *p* = 0.0038, aCSF vs. aCSF+BSA; *p* = 0.001 aCSF vs. aCSF+HSA. (**D**) Zinc release to the microdialysate of MCAo rats between 2 and 5 h post-injury. The change in zinc release levels in the microdialysate were plotted and the individual AUC was analyzed, indicating significance *p* = 0.0006, aCSF vs. aCSF+HSA; *p* = 0.0001, aCSF+BSA vs. aCSF+HSA. (**E**) Lactate release to the microdialysate of MCAo rats between 2 and 5 h post-injury. The change in lactate release levels in the microdialysate was plotted and the individual AUC was analyzed, showing significance at *p* = 0.0482, aCSF vs. aCSF+HSA. Data from the AUC are the mean ± SEM. A significant difference was accepted at *p* < 0.05 by one-way ANOVA followed by Holm–Sidak’s test. (**F**) Neurological deficit scores in MCAo rats (n = 10, 9, 7 rats for aCSF, aCSF+BSA, aCSF+HSA, respectively); 7 days behavioral significance at *p* = 0.0075, aCSF vs. aCSF+BSA; *p* = 0.0095, aCSF vs. aCSF+HSA. (**G**) Grasping power, which was evaluated by grip test on right forelimbs (unaffected side) and left forelimbs (stroke-affected side) in MCAo rats (n = 9, 9, 7 rats for aCSF, aCSF+BSA, aCSF+HSA, respectively); 7 days behavioral significance at *p* = 0.0121, aCSF vs. aCSF+BSA; *p* = 0.0387, aCSF vs. aCSF+HSA. Behavioral data were analyzed by two-way ANOVA followed by Holm–Sidak’s test. (**H**) Representative micrographs of NeuN- or ED1-immunoreactive cells in mid-infarct cortex, shown by a red square in the subfigure, at 1 week after injury. Scale bar: 100 μm. (**I**) Histogram for quantitation of NeuN-IR density in the panel H images; n = 6, 3 rats for aCSF, aCSF+HAS, respectively; significance at *p* = 0.0143 by one-way ANOVA followed by Holm–Sidak’s test. (**J**) Histogram for quantitation of ED1-IR density in the panel H images. Data are expressed as the mean ± SEM.

We presented data showing an effective intervention for injury-induced brain damage and behavioral impairment using BSA-supplemented aCSF-microdialysis. We further tested if supplementation of aCSF with human serum albumin (HSA) could be as neuroprotective as BSA supplementation in MCAo rats. Figure 2A,B show that microdialysis treatment with aCSF+HSA or with aCSF+BSA significantly reduced ischemia-induced infarction (*p* < 0.05), compared to treatment of microdialysis with aCSF only. Excessive glutamate release leads to excitotoxicity, which has a prominent role in ischemic brain injury. We thus analyzed the levels of glutamate, zinc ions, and lactate in the microdialysate of aCSF-, aCSF+BSA-, or aCSF+HSA-treated MCAo rats between 2 and 5 h post-injury. MCAo rats subjected to aCSF treatment released constant levels of glutamate and lactate, but not zinc ion, to the microdialysate (Figure 2C–E). Significantly more glutamate was recovered (or removed from the ischemic core) in the microdialysate of aCSF+BSA- or aCSF+HSA-treated MCAo rats, compared to that in aCSF-treated MCAo rats (all *p* < 0.01, Figure 2C(ii)). Interestingly, significantly more zinc was recovered in the microdialysate of aCSF+HSA-treated MCAo rats, compared to that of aCSF- or aCSF+BSA-treated MCAo rats (all *p* < 0.01, Figure 2D(ii)). However, three groups of MCAo rats released constant and similar levels of lactate to the microdialysates within 135 min of microdialysis (Figure 2E). Beginning at 165 min of microdialysis, the aCSF+HSA-treated MCAo rats had more lactate in the dialysate than the aCSF+BSA and aCSF-treated MCAo rats (*p* < 0.05, Figure 2E(ii)). Figure 2F shows that both aCSF+BSA and aCSF+HSA treatments effectively attenuated injury-induced functional deficits, at 7 days post-injury (*p* < 0.05 or 0.01). As expected, there was no difference between the effects of aCSF+BSA and aCSF+HSA. Figure 2G shows the grasping power of experimental rats in which aCSF+BSA and aCSF+HSA treatment significantly promoted recovery, at 7 days post-injury (*p* < 0.05). Histological assessment of aCSF+HSA-treated MCAo rat brains showed that fewer ED1-IR cells and more NeuN-IR cells (*p* < 0.05) were observed in the ipsilateral cortex of aCSF+HSA-treated rats compared to those in aCSF-treated rats (Figure 2H). Thus, tMD with aCSF+BSA or aCSF+HSA effectively removed stimulants and reduced neuronal damage and brain infarction in MCAo rats (Figure 2I,J).

### 2.3. Protective Effect of tMD at the Golden 6th Hour Post-Ischemic Injury

**Figure 3:**
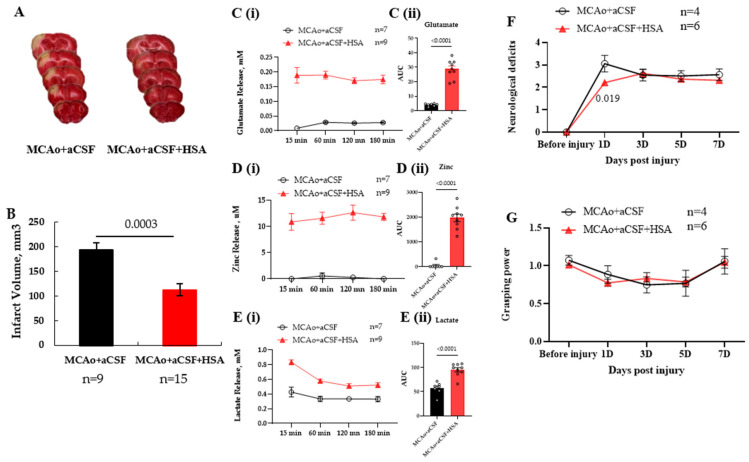
Effect of intervention at 6 h post-injury with tMD on brain infarction, secreted stimulants, and behavioral performance in MCAo rats. (**A**) Representative TTC-staining images of brain slices from MCAo rats of various groups at 1 week post-injury. (**B**) Brain infarcted volume (mm^3^) in ischemic rats (n = 9, 15 rats for aCSF, aCSF+HSA, respectively); significance at *p* = 0.0003. (**C**) Glutamate release to the microdialysate of MCAo rats between 6 and 9 h post-injury (n = 7, 9 rats for aCSF, aCSF+HSA, respectively). The change of glutamate release in the microdialysate was plotted as a line chart (left panel) and the area under the curve (AUC) was analyzed (bar chart, right panel, with symbol of individual data). Quantification of the AUC shows significance at *p* < 0.0001, aCSF vs. aCSF+HSA. (**D**) Zinc release to the microdialysate of MCAo rats between 6 and 9 h post-injury (n = 7, 9 rats for aCSF, aCSF+HSA, respectively). The change in zinc release levels in the microdialysate was plotted and the AUC was analyzed with a significant result, *p* < 0.0001, aCSF vs. aCSF+HSA. (**E**) Lactate release to the microdialysate of MCAo rats between 6 and 9 h post-injury (n = 7, 9 rats for aCSF, aCSF+HSA, respectively). The change in lactate release in the microdialysate was plotted and the AUC was analyzed with significance at *p* < 0.0001, aCSF vs. aCSF+HSA. Data are the mean ± SEM. A significant difference was accepted at *p* < 0.05 by one-way ANOVA followed by Holm–Sidak’s test. (**F**) Neurological deficit scores in rats surviving one week after cerebral ischemia (n = 4, 6 rats for aCSF, aCSF+HSA, respectively). Day one behavioral significance at *p* = 0.019, aCSF vs. aCSF+HSA. (**G**) Grasping power by grip test on unaffected forelimbs and stroke-affected forelimbs in MCAo rats (n = 4, 6 rats for aCSF, aCSF+HSA, respectively). Behavioral data were analyzed by the generalized estimating equation (GEE) in which adjustments are made within or between groups. Data are expressed as the mean ± SEM.

To test the therapeutic efficacy of tMD at a longer time point after injury, we conducted a therapeutic intervention on ischemia at 6 h post-injury. Application of tMD at 6 h post-injury significantly reduced brain infarction in MCAo rats (Figure 3A,B). Analysis of the microdialysate collected within 6–9 h post-injury shows that tMD treatment removed significant levels of glutamate, zinc ion, and lactate to the microdialysate and thus conferred protection (all *p* < 0.01, compared to aCSF treatment; Figure 3C–E). Figure 3F demonstrates that tMD treatment tended to reduce neurological deficits at 1 day post-injury in MCAo rats compared to that of MCAo + sham or MCAo + aCSF rats. However, this tMD treatment did not affect the grasping power of the MCAo rats (Figure 3G). Supplementary Tables S1 and S2 show the changes in behavior of the rats when the microdialysis was performed at 2 vs. 6 h.

## 3. Discussion

Stroke-induced brain injury could result in severe local inflammation and irreversible neuronal death. During the first few hours after ischemic stroke, numerous harmful molecular events contribute to injury. In this study, we inserted a minimally invasive sampling probe into the ischemic core and perfused it with an oncotic agent-based buffer to intentionally induce a fluid shift, allowing for the timely removal of stimulants (toxicant). Importantly, we identified an unanticipated role of serum albumin-supplemented buffer in the removal of toxicants from the ischemic core. In the first part of this study, we demonstrated that both BSA and HSA in aCSF buffer, used in tMD therapy, significantly reduced the volume of cerebral infarction, promoted cell survival, and substantially improved behavioral function in animals with ischemic stroke. BSA is derived from cattle blood, while HSA is derived from human plasma. BSA is commonly used in laboratory settings as a protein standard in assays [22]. We initially discovered that BSA-supplemented aCSF in tMD therapy protected the brain from ischemic injury. However, because BSA is more likely to trigger an immune response when introduced into humans, its use in human therapies is limited. HSA, being native to humans, has low immunogenicity, making it preferable for medical applications, particularly in therapies such as volume expansion and the treatment of hypoalbuminemia [23]. Therefore, we further investigated the neuroprotective effect of HSA-supplemented aCSF in the tMD buffer on rats subjected to ischemic stroke. In the second part of the study, we further demonstrated the existence of a broad therapeutic window of neuroprotective tMD therapy, showing that treatment initiated even 6 h after the onset of ischemia was highly effective. This finding suggests that the treatment remains effective even when administered during the late stages of the ischemic cascade. Such a wide therapeutic window has significant translational relevance, as most patients receive medical attention several hours after stroke onset, at which point thrombolysis with rt-PA, the only approved treatment for acute stroke, is no longer safe or effective [24,25].

After ischemic stroke, various damage-associated molecules are released from the ischemic core and diffuse to the ischemic penumbra, activating microglia and promoting proinflammatory responses that may cause damage to the local tissue [26]. Glutamate excitotoxicity is a major mechanism that kills neurons after stroke [27,28,29,30]. Elevated extracellular glutamate reflects excessive neuronal excitation or signs of severe cell damage resulting in the release of intracellularly stored glutamate. Moreover, ischemia-induced excess glutamate and oxidative stress compromise the blood–brain barrier (BBB), leading to increased vascular permeability and immune cell infiltration [30,31]. The ischemic core and penumbra must be treated early to prevent further damage. This is the first time that we used conventional microdialytic devices for therapeutic use in cerebral ischemia. We perfused the microdialysis catheter with an oncotic agent containing solution to intentionally produce a fluid shift to remove stimulant and reduce brain injury. There was a sustained and significant (all *p* < 0.01) increase in glutamate and zinc levels in the microdialysate of the tMD group, leading to reduced excitotoxicity and brain damage. More importantly, tMD, even applied at 6 h after stroke, removed significantly higher levels of glutamate and zinc ions (all *p* < 0.01) from the injured brain and strongly protected against ischemic brain damage. Free zinc ions and glutamate have been reported to be released together in brain ischemia, providing the first demonstration of co-release of glutamate and Zn^2+^ during ischemia and reperfusion [32]. Consistent with this result, we showed the co-presence of zinc ions and glutamate in the microdialysate of MCAo rats. Furthermore, much higher levels of zinc ions and glutamate were rinsed off by tMD either between 2 and 5 h or 6 and 9 h post-ischemia and reperfusion (Figure 2D,E and Figure 3D,E). Concurrently, a lower degree of brain injury and sustained neurological restoration were observed in tMD-treated MCAo rats (*p* < 0.01 or 0.05). By removing excess glutamate, tMD helps stabilize the BBB and reduces calcium influx, mitochondrial dysfunction, and oxidative stress, thereby maintaining intracellular signaling homeostasis and preserving pro-survival pathways and maintains synaptic plasticity mechanisms essential for recovery. Furthermore, elevated zinc concentrations within the synaptic cleft of ischemic neurons have been associated with cell death, suggesting that zinc toxicity could represent an independent risk factor for ischemic stroke [33]. tMD facilitates the removal of excess zinc, and the maintenance of zinc homeostasis reduces oxidative damage and stabilizes neuronal metabolic processes. In summary, tMD exerts neuroprotective effects by reducing excitotoxicity, stabilizing metabolic and inflammatory responses, and restoring neurological function. These mechanisms contribute to preserved synaptic activity, enhanced neuronal survival, and potential functional recovery following ischemic stroke. This finding suggests that tMD potentially modified the hostile environment associated with the secondary cell death of the ischemic brain and is thus protective.

Lactate levels in the microdialysate were analyzed to show the status of energy metabolism in experimental rats. Lactate release in the first 2 h of microdialysis was similar among aCSF-treated, aCSF-BSA-treated, and aCSF-HSA-treated MCAo rats, indicating similar degrees of energy impairment (Figure 2E). Beginning at the third hour of microdialysis (i.e., 5 h post-stroke) through 9 h post-stroke, a significant increase in lactate was shown in aCSF-HSA-treated microdialysate (Figure 2E and Figure 3E). Increased lactate release exhibits an energetic deficit. Extracellular accumulation of lactate may cause acidosis and is associated with increased mortality. Because the ischemic core refers to the most severely damaged region due to an insufficient blood supply, thus leading to irreversible cell death within minutes, therapeutic interventions primarily aim to preserve the penumbral area. In this study, the dialysis probe was placed targeting the ischemic core, minimizing potential damage associated with probe insertion. There was no obvious change in TTC staining in the region of probe insertion, as shown in Supplementary Figure S1. This indicates a minimally invasive surgery. Our results indicate that controlled solute/stimulant extraction from the ischemia core center can be performed and can lead to increased survival compared with craniectomy only. The present study provides the first evidence that necrotic core exudates can be targeted to limit stroke injury and suggests that tMD may represent a novel post-stroke therapy.

The present study demonstrates that microdialytic delivery of BSA or HSA induced sustained neurological recovery after focal cerebral ischemia in rats. BSA is chemical grade for research use, whereas HSA is diagnostic grade of ultra-high purity that has been extensively tested for a full panel of safety and analytical data. We also checked the purity of HSA and BSA in spinal cord cultures and the result, shown in Supplementary Figure S2, is consistent with this suggestion. To evaluate the therapeutic efficacy of tMD at a later time point following injury, we employed therapeutic intervention to rat brain at six hours post-ischemia. The application of tMD at six hours post-injury significantly reduced brain infarct volume in MCAo rats. Analysis of microdialytic elutes collected between six and nine hours post-injury indicated that tMD treatment effectively removed significant levels of glutamate, zinc ions, and lactate. These findings suggest that the application of tMD within six to nine hours after ischemic stroke may still exert therapeutic benefits by facilitating the removal of neurotoxic substances. The present study is preclinical animal research with translational potential. Because rat and human brains differ greatly, many practical challenges remain to be solved. These include optimizing probe design for human use, determining the optimal duration of treatment, ensuring long-term safety in patients, etc.

The ischemic core represents the region with critically reduced cerebral blood flow (CBF) and metabolic oxygen consumption, resulting in irreversible tissue damage following stroke. The penumbral region surrounding the ischemic core remains potentially salvageable. The imaging analysis suggests that the specific location of the salvaged penumbra is of greater clinical significance than its overall volume. Notably, the preservation or loss of ischemic penumbral areas in proximity to the ischemic core may play a critical role in determining the extent of residual disability in patients with acute MCA stroke. In cases of middle cerebral artery (MCA) stroke, infarct progression occurs as the ischemic core gradually expands into the penumbra within the initial hours post-stroke [34]. In this study, although we found that infarct volume was reduced following the application of tMD at 6 h post-injury, there was no long-term improvement in either grasping power or neurological deficits observed with HSA treatment (Figure 3). Therefore, we suggest the application of tMD as soon as possible after ischemic stroke, not only to reduce cerebral infarction but also improve functional recovery.

This study assessed neuroprotection within seven days post-injury. Future long-term investigations will be conducted to determine whether the short-term neuroprotective effects observed translate into sustained functional recovery and to evaluate the potential for any delayed adverse effects. In the present study, we put albumin in the dialysate to create oncotic pressure for resolving brain edema. **Oncotic** pressure is a form of osmotic pressure exerted by proteins in a blood vessel’s plasma that usually tends to pull water into the circulatory system. Most oncotic pressure in our microdialytic buffer is generated by the inclusion of high quantities of albumin (10%). Because albumin cannot escape through the semipermeable membrane of the microdialytic probe, the oncotic pressure of the microdialytic buffer tends to draw water accompanied by stimulants (or toxicants) into the probe. A study conducted in test tubes (in vitro) by Trickler and Miller [35] showed that BSA in the perfusate could increase protein recovery by 10–25%. Albumin within the microdialysis catheter may directly bind cytokines or proteins that cross the membrane into the catheter. Agreeing with this, we observed reduced numbers of activated microglia/macrophage in the ischemic cortex of groups receiving the artificial CSF with BSA or HSA treatment. This approach may contribute to reducing local tissue inflammatory cytokine levels in the penumbra. Therefore, tMD may represent a promising stroke treatment. The present study provides clinically relevant evidence warranting rapid proof-of-concept studies in stroke patients.

## 4. Materials and Methods

### 4.1. Materials

Microdialysis probes (CMA 12 Elite probe 4 mm) were from CMA (Holliston, MA, USA). Artificial cerebrospinal fluid (aCSF) was from Harvard Apparatus (59-7316, Holliston, MA, USA). Bovine serum albumin (BSA) was from Affymetrix (Cas#9048-46-8, Santa Clara, CA, USA) Cohn Fraction V. Human serum albumin (HSA) in powder form was from Access Biologicals (Vista, CA, USA), whereas HSA in 20% solution (designated as NCU-20) was from Behring (Danville, CA, USA). Hank’s balanced salt solution (HBSS) was from Gibco (Waltham, MA, USA). Fibrin glue was from Beriplast P (Marburg, Germany). Other reagents were purchased from Sigma-Aldrich (St. Louis, MO, USA), unless stated otherwise. All chemicals used were of analytical grade or higher quality.

### 4.2. Surgical Procedure and Treatment

Adult male Long–Evans (LE) rats, weighing 250–350 g and bred in the National Laboratory Animal Breeding and Research Center, Taipei, were used. All animal procedures were approved by the Institutional Animal Care and Use Committee (IACUC) at Taipei Veterans General Hospital under the reference IACUC 2013-071 and 2023-148. Rats were anesthetized in an induction chamber ventilated with 5% isoflurane gas and maintained on anesthesia with 1.5% isoflurane using a nose mask. Focal ischemic infarct was induced in the territory of the middle cerebral artery (MCA) in the right cerebral cortex of the LE rats, as previously described [36,37,38]. Briefly, the right MCA was ligated with 10-0 monofilament nylon ties. Both common carotid arteries were occluded by micro-aneurysm clips for 1 h. Reperfusion of flow was confirmed visually during surgery before closure of the wound. At 2 or 6 h after ischemia-reperfusion, the animal’s head was secured in a stereotaxic frame. After a midline skin incision, a burr hole was made on the skull with the coordinates of 0 mm posterior and 5.5 mm lateral to the bregma. A microdialysis probe (CMA 12; 20 kDa cut-off), which had been equilibrated with perfusion buffer (or plus treatment) was implanted through the burr hole to the ischemic core (4 mm below the dura surface). This target brain region, the ischemic core, was determined using an atlas of the rat brain and by our experience in studying cerebral ischemia [39]. An infusion pump was then prompted to perfuse buffer (or plus treatment) continuously for 3 h at a flow rate of 5 μL/min. The efflux from the microdialysis probe was collected at 15 min intervals for analyzing the biochemical contents. Animals were allowed to recover from surgery, and the skin wounds were closed. Behavioral evaluations of experimental rats, including neurological function and grasping tasks, were conducted at 1, 3, 5, and 7 days post-injury. At one week after ischemia, animals were euthanized with an overdose of pentobarbital (200 mg/kg) injection for morphological assays.

### 4.3. Neurological Deficit Score (NSD) Test

A five-point grading scale of the NSD test was used to assess motor function with overall observation [40,41]. A higher value represents more severe motor deficit and vice versa. Five categories were scored: 0, normal motor function or no apparent deficits; 1, contralateral forelimb flexion; 2, decreased grip of contralateral forelimb while the tail was pulled; 3, spontaneous movement in all directions, contralateral circling if pulled by the tail; 4, spontaneous contralateral circling; and 5, no spontaneous motor activity or death. Each rat was given a value (0~5) at 1, 3, 5, or 7 days after MCAo surgery.

### 4.4. Grasping Power Test

Contralateral motor deficits in the rat forelimbs due to damage of the stroke-affected brain were evaluated by the grasping power test using a commercial grip-strength meter (Grip-strength meter 303500, TSE systems Corp., Midland, MI, USA) for rats [36,37]. Briefly, rats were placed over a Perspex plate in front of a grasping trapeze. By pulling their tail, we impelled the rats to instinctively grab anything they could (in this case, the trapeze) to stop their involuntary backward movement. When our pulling force overcame its grip-strength, the animal lost its grip on the trapeze. The preamplifier of the grip-strength meter showed a resulting peak pull force that was used to represent the grasping power of the tested limb. To ensure accuracy, we performed at least 10 trials per rat in each of the grasping power tests, and the three highest grasping powers were recorded in each case.

### 4.5. Morphological Analysis

For infarct volume analysis, rat brains were quickly removed, placed in a sectioning apparatus (Zivic Miller, Zelienople, PA, USA), and sectioned into 2 mm coronal slices. The resulting slices were stained with 2% 2,3,5-triphenyl tetrazolium chloride (TTC) for 30 min and fixed in 10% buffered formalin solution overnight. TTC positive staining, indicating viable tissues, is used to verify successful stroke and treatment. Cerebral infarction volume (negative TTC stain area) was quantified using digital imaging and analyzed with image analysis software (National Institutes of Health Image J software, version 1.42) [35]. Notably, each brain slice was calculated in the form of delta of the bilateral viable tissue (red portion) with dying tissue (white portion). Infarct volume (mm^3^) was calculated by determining the infarction area in each 2 mm slice, followed by summing the volumes of all slices for each subject. For fluorescence immunocytochemical staining, the brain tissues were post-fixed with 4% paraformaldehyde, processed in a series with 15% and 30% sucrose, and finally embedded in OCT compound (Sakura Fine Technical, Tokyo, Japan). Tissues were cut into serial 20 μm sections with a cryostat. Immunocytochemical staining was performed on serial sections, as previously described [42,43]. The brain section surrounding the ischemic core of MCAo rats was processed for immunohistochemical (IHC) staining for neuronal markers (by anti-NeuN, Millipore, Burlington, MA, USA), activated microglia/macrophage (by anti-ED-1 (CD68, Bio-Rad, Hercules, CA, USA)), and astroglia (by anti-GFAP, Chemicon International, Temecula, CA, USA).

### 4.6. Western Blot Analysis

After experimental periods, brain tissues were homogenized in lysis buffer containing 40 mM Tris buffer (pH 7.5), 8 M urea, 4% CHAPS, 1 mM PMSF, 1 mM Na_3_VO_4_, 1 mM dithiothreitol, and a protease inhibitor kit (BM, Eiterfeld, Germany). Aliquots of cell lysates (5 μg protein/lane) were analyzed by Western blot analysis using 8~12% SDS-PAGE, as previously described [44,45]. The resulting PDVF membranes were probed with antibodies against GAP43 (Chemicon International, Temecula, CA, USA), also named neuromodulin; newly synthesized neurites, MAP-2 (microtubule associated protein 2, Millipore, Darmstadt, Germany), a neuronal marker, and ED1 (CD68; activated microglia/macrophage); LC3A (Novus Biologicals, Centennial, CO, USA), a microtubule-associated protein 1A/1B-light chain 3 in autophagy-related processes; AIF (apoptosis inducing factor); and actin (Santa Cruz Biotechnology, Dallas, TX, USA).

### 4.7. Biochemical Assays

Levels of released glutamate, lactate, and zinc ion in the microdialysate were analyzed using commercial kits. They included a kit from Biovision K629-100 for glutamate, a kit from Biovision K387-100 for zinc ion, and a kit from Eton Bioscience Inc. (San Diego, CA, USA) for lactate.

### 4.8. Statistical Analysis

One-way ANOVA (analysis of variance) followed by Holm–Sidak’s test was used to determine significance. Two-way ANOVA with the Geisser–Greenhouse correction and Holm–Sidak’s multiple comparisons test was performed with individual variances computed for each comparison. Rat behavioral data were analyzed by the generalized estimating equation (GEE) in which adjustments are made within or between groups. A significant difference was accepted at *p* < 0.05.

## 5. Conclusions

The present study shows that albumin-supplemented aCSF in the tMD buffer affords high-grade neuroprotection in focal cerebral ischemia. To our knowledge, this is the first demonstration that provides a method for site-specific microtherapy. Importantly, tMD initiated as late as 6 h after stroke onset reduced infarct volume and improved behavioral scores. This 6 h time frame is clinically relevant in that it is logistically difficult to formulate therapy in many patients with stroke at earlier times. This tMD offers great promise in the therapy of cerebral ischemia. It may be appropriate to consider early-phase clinical trials in patients with ischemic stroke, perhaps without major side effects. tMD stands as a promising therapy for stroke.

## Data Availability

The data underlying this article are available in the article and the datasets analyzed during the current study are available from the corresponding author on reasonable request.

## Supplementary Materials

The following supporting information can be downloaded at: https://www.mdpi.com/article/10.3390/ijms26083821/s1.

## Abbreviations

Therapeutic microdialysis (tMD), neurological deficit score (NSD), middle cerebral artery (MCA); 2,3,5-triphenyl tetrazolium chloride (TTC).
