# Supplementary material for: Targeting the Ischemic Core: A Therapeutic Microdialytic Approach to Prevent Neuronal Death and Restore Functional Behaviors
Source: Int J Mol Sci. 2025 Apr 17;26(8):3821. doi: 10.3390/ijms26083821 (PMC12027531; doi:10.3390/ijms26083821)
Supplement: Supplementary file 1 [file ijms-26-03821-s001.zip › ijms-3503715-supplementary.pdf]

Supplementary Materials

Supplementary Figure S1, Coronal sections of normal-Sham rat brain after conducting 2,3,5-triphenyl tetrazolium chloride (TTC) staining

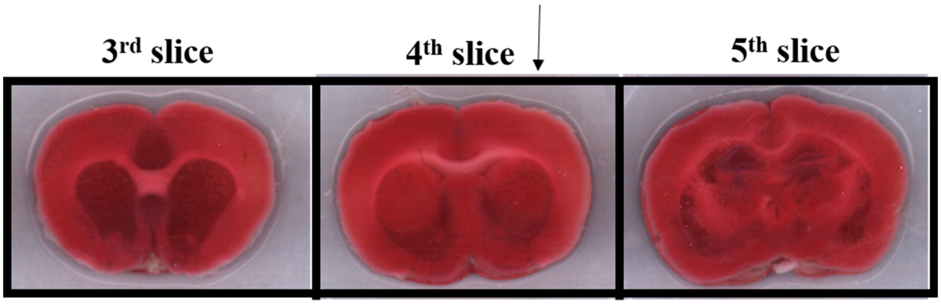

Microdialysis probe was inserted to normal rat brain for three hours, the probe was then removed. Seven days after probe removal, brain was sliced into 8 serial 2-mm sections and subjected to TTC staining. Note that arrow in slice 4 contains the site of probe insertion (0 mm posterior and 5.5mm to the bregma).

Supplementary Figure S2

Effects of rat brain microdialysate on adult spinal cord glial cultures (A) adult glial cells grown in 48 well plates (B) adult glial cells grown in 24well plates.

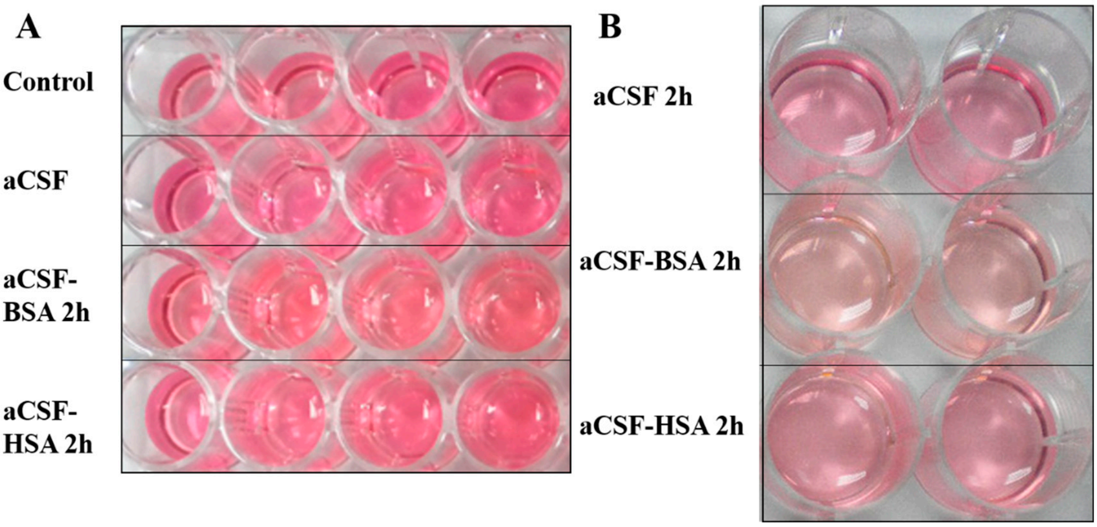

Microdialysate of the 2nd ,4th ,6th ,8th tubes eluted from MCAo rats were collected and pooled. Aliquot of 20ul microdialysate /500ul medium/well was added to adult spinal cord cultures maintained in DMEM+4%FBS and further incubated for two days. Note that change (more acidic) of medium color was found in BSA-MCAo treated cultures but not in others.

All microdilysates did not change the color of cultured medium when they were first added to culture. Two days after treatment, obvious color change (more acidic) in medium of BSA-MCAo treatment but not in others. This indicate more lactate levels was released from these cells. Glial cells are active in glutamate uptake and also tolerant to high zinc levels. In the process of aerobic glycolysis, glucose is metabolized to pyruvate and further converted into acetyl-coenzyme A, which is used for mitochondrial energy production. Under conditions of mitochondrial dysfunction, pyruvate is fermented into lactate. The unknown impurities in BSA-microdialysate may impair mitochondrial function.

Materials and Method

Adult spinal cord glial cultures: Sprague-Dawley (SD) rats were obtained from the Animal Center of the National Yang Ming Chiao Tung University, Taiwan. Glial cultures were prepared from the adult spinal cord of SD rats by mechanical dissociation, according to the methods described by Tsai and Lee (Fay et al., 2023). Cultures were maintained in Dulbecco's modified Eagle's medium (DMEM, Invitrogen) supplemented with 10% FBS, Invitrogen) at 37 ◦C in an incubator with a water-saturated atmosphere of 5% CO2/95% air. After 10–14 days in vitro, confluent cultures were treated with microdialysate and further incubated for 2 days.

Table S1. The quantified rat behaviors of MCAo rats with therapeutic microdialysis conducted at 2 vs 6 hours post-injury.

		neurological deficit (nd)	group 1	group 2	grasping	group 1	group 2
		significance (p value)	mean (aCSF)	mean (HSA)	significance (p value)	mean (aCSF)	mean (HSA)
MCAo-2hr-microdialysis	Before injury		0	0		1.10	0.96
	1D	0.461	3.10	2.89	0.007	0.49	0.71
	3D	0.260	2.83	2.51	0.255	0.68	0.69
	5D	0.073	2.71	2.19	0.044	0.71	0.84
	7D	0.021	2.54	1.87	0.006	0.68	0.92
MCAo-6hr-microdialysis	Before injury		0.00	0.00		1.07	1.01
	1D	0.019	3.05	2.21	0.771	0.89	0.77
	3D	0.830	2.54	2.62	0.442	0.75	0.83
	5D	0.679	2.51	2.36	0.677	0.77	0.79
	7D	0.473	2.56	2.31	0.793	1.06	1.05

Behavioral data of neurological deficits and grasping power were analyzed by Generalized Estimating Equation (GEE) in which adjustments are made within or between group.

Table S2. The quantified rat behaviors of MCAo rats with therapeutic microdialysis conducted at 2 vs 6 hours post-injury.

Miocrodilaysis		neurological deficit	grasping
hour (post MCAo)		p value	p value
2	group	0.000	0.035
	time	0.000	0.000
	group x time	0.164	0.028
6	group	0.039	0.786
	time	0.000	0.004
	group x time	0.084	0.855
